# Comparative Lipidomics of *Caenorhabditis elegans* Metabolic Disease Models by SWATH Non-Targeted Tandem Mass Spectrometry

**DOI:** 10.3390/metabo5040677

**Published:** 2015-11-11

**Authors:** Jeevan K. Prasain, Landon Wilson, Hieu D. Hoang, Ray Moore, Michael A. Miller

**Affiliations:** 1 Department of Pharmacology and Toxicology, University of Alabama at Birmingham, Birmingham, AL 35294, USA; E-Mail: jprasain@uab.edu; 2 Targeted Metabolomics and Proteomics Laboratory, University of Alabama at Birmingham, Birmingham, AL 35294, USA; E-Mails: empy1977@uab.edu (L.W.); raymoore@uab.edu (R.M.); 3 Department of Cell, Developmental and Integrative Biology, University of Alabama at Birmingham, Birmingham, AL 35294, USA; E-Mail: hdhoang@uab.edu

**Keywords:** *Caenorhabditis elegans*, lipidomics, SWATH, tandem mass spectrometry, insulin, prostaglandin, cyclooxygenase, triglycerides, arachidonic acid, MS/MS^ALL^

## Abstract

Tandem mass spectrometry (MS/MS) with Sequential Window Acquisition of all Theoretical (SWATH) mass spectra generates a comprehensive archive of lipid species within an extract for retrospective, quantitative MS/MS analysis. Here we apply this new technology in *Caenorhabditis elegans* (*C. elegans*) to identify potential lipid mediators and pathways. The DAF-1 type I TGF-β and DAF-2 insulin receptors transmit endocrine signals that couple metabolic status to fertility and lifespan. Mutations in *daf-1* and *daf-2* reduce prostaglandin-endoperoxide synthase (*i.e.*, Cox)-independent prostaglandin synthesis, increase triacylglyceride storage, and alter transcription of numerous lipid metabolism genes. However, the extent to which DAF-1 and DAF-2 signaling modulate lipid metabolism and the underlying mechanisms are not well understood. MS/MS^ALL^ with SWATH analysis across the groups identified significant changes in numerous lipids, including specific triacylglycerols, diacylglycerols, and phosphatidylinositols. Examples are provided, using retrospective neutral loss and precursor ion scans as well as MS/MS spectra, to help identify annotated lipids and search libraries for lipids of interest. As proof of principle, we used comparative lipidomics to investigate the prostaglandin metabolism pathway. SWATH data support an unanticipated model: Cox-independent prostaglandin synthesis may involve lysophosphatidylcholine and other lyso glycerophospholipids. This study showcases the power of comprehensive, retrospectively searchable lipid archives as a systems approach for biological discovery in genetic animal models.

## 1. Introduction

**Figure 1:**
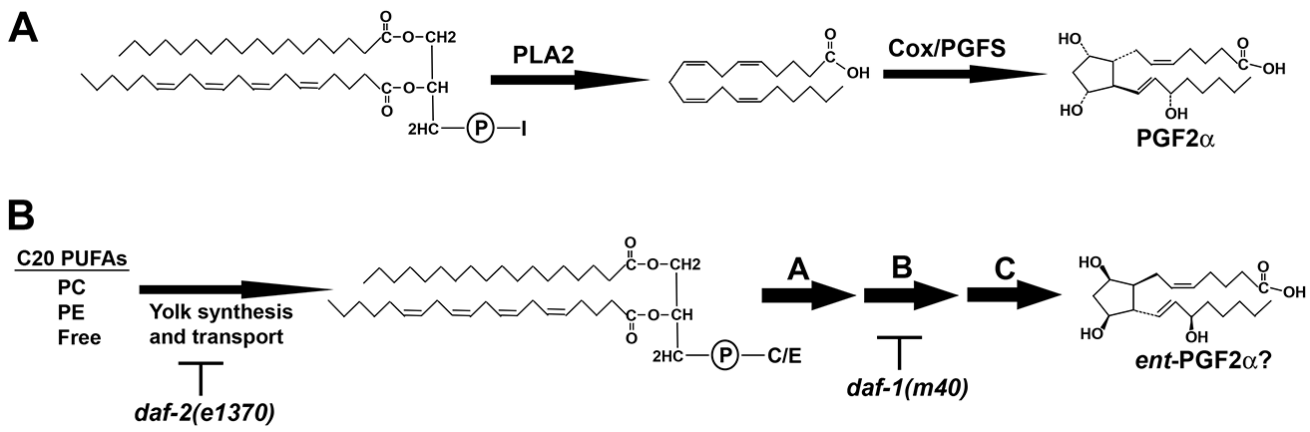
Prostaglandin biosynthesis pathways. (**A**) In the Cox pathway, phospholipase A2 cleaves arachidonic acid (AA) from the *sn-2* position of phospholipids like PI, generating lysoPI species lacking AA. PGF2α is synthesized from free AA by the sequential actions of Cox and prostaglandin F synthase (PGFS); (**B**) In *C. elegans*, C20 PUFAs are delivered to oocytes in yolk lipoprotein complexes. Yolk PUFAs are primarily found esterified to PC and PE, and in free forms. An incompletely understood metabolic pathway in oocytes, shown arbitrarily as steps A, B, and C, converts yolk lipids into F-series prostaglandins, such as *ent-*PGF2α (or a co-eluting stereoisomer). I, inositol; C, choline; E, ethanolamine; PC, phosphatidylcholine; PE, phosphatidylethanolamine; C20, 20-carbon.

Lipids are a structurally diverse group of naturally occurring amphipathic molecules that include fatty acids, glycerides, phospholipids, sphingolipids, sterols, prenols, and waxes. They are principal components of cellular membranes, store energy in the form of triacylglycerides, and function as signaling molecules. Ketoacyl and isoprene building blocks form lipid backbones, which can be coupled to sugars, amines, and other small molecules. For example, the glycerophospholipid phosphatidylinositol contains two esterified fatty acids at the glycerol *sn-1* and *sn-2* positions and an inositol sugar head group at *sn-3*. 20-carbon (C20) polyunsaturated fatty acids (PUFAs) are liberated from *sn-2* by phospholipase A2 and oxidized into prostaglandins and other eicosanoids [1,2]. Prostaglandin-endoperoxide synthase (a.k.a. cyclooxygenase or Cox) catalyzes cyclopentane ring formation (Figure 1A). Through enzymatic cascades, lipids are converted from one structural form to another, comprising pathways for modifying cell structure and function.

The lipidome is the complete lipid composition of a tissue or organism, which can contain well over 10,000 different species. As a systems biology approach, lipidomics aims to determine and quantify this composition. Mass spectrometry is a widely used analytical platform for lipidomics because of its sensitivity, specificity, and accuracy [3,4,5]. In contrast to targeted approaches that measure a set of predetermined lipids, untargeted lipidomics seeks to extend coverage to all detectable lipid species. This untargeted approach is greatly strengthened by high-resolution mass spectrometers that help discriminate among isobaric compounds, which have the same nominal mass, but different molecular formula. The “top-down” method is solely reliant on accurate mass detection of the parent lipid. It is unbiased toward lipid class, but biased toward the most abundant species. On the other hand, the “bottom-up” method relies on detection of structure-specific breakdown products using tandem mass spectrometry (MS/MS). It is biased toward lipid class, but capable of detecting species with low abundance. A major disadvantage of MS/MS is the targeted nature: one must anticipate which lipid classes are best candidates *a priori*.

MS/MS with data-independent Sequential Window Acquisition of all Theoretical (SWATH) mass spectra provides a powerful combination of top-down and bottom-up methods [6,7]. Data-independent SWATH collects MS/MS spectra in 1 *m*/*z* windows across the entire mass range simultaneously. SWATH using the AB SCIEX 5600 triple time-of-flight (TOF) mass spectrometer creates a comprehensive high mass resolution lipid library. This technology offers several strengths for comparative lipidomics between wild-type and mutant animals. Major strengths include: (1) it does not require *a priori* assumptions; (2) library data are quantitative (relative); and (3) high mass resolution and MS/MS enable identification of many lipids. A particularly important feature is that the data can be searched retrospectively, eliminating the need to repeat sample preparation and analyses every time there is a new lipid of interest. For example, SWATH can be used to generate lipid libraries for rare or difficult to collect wild-type or mutant animal tissues. Researchers can then search the libraries using objective criteria, relying on software-based lipid annotations, or targeted approaches using neutral loss or precursor ion scans. The libraries provide a data-mining resource to generate testable models for biological discovery.

The nematode model *C. elegans* is well suited for comprehensive lipidomics, although most studies have relied on data-dependent, targeted strategies [8,9]. Genetic screens and genome-editing technologies can be used to identify lipid-modifying enzymes or gene products impacting lipid metabolism [10,11,12]. The DAF-1 type I TGF-β and DAF-2 insulin receptors transmit endocrine signals that promote reproduction and modulate lifespan [13,14]. Lipids appear to be central to multiple *daf-1* and *daf-2* functions, although the mechanisms are not well understood. Genetic loss of either signaling pathway causes altered transcription of lipid metabolism genes, triacylglyceride (TAG) accumulation, and ovarian prostaglandin deficiency [15,16,17,18,19]. In the adult ovary, oocytes secrete multiple F-series prostaglandins that stimulate sperm motility (Figure 1B) [20,21]. DAF-1 and DAF-2 promote prostaglandin synthesis through a metabolic pathway lacking Cox enzymes [15,16]. Liquid chromatography electrospray ionization tandem mass spectrometry (LC-MS/MS) data from mouse and zebrafish tissues, including *Cox-1*; *Cox-2* double knockout mice provide evidence that this pathway is conserved [16,20]. However, the biochemical steps between arachidonic acid (AA) and PGF2 formation are not well understood.

Here we use MS/MS^ALL^ with SWATH analysis in *C. elegans* to evaluate the utility of comprehensive, searchable lipid libraries as a biological discovery tool. We provide selected examples for validating and searching the libraries. Disrupting *daf-1* and *daf-2* signaling causes specific alterations in numerous lipids, including TAG, phosphatidylcholine (PC), and phosphatidylinositol (PI) species. These lipids are potential downstream effectors and candidate markers to assess signaling activity. To investigate an incompletely understood metabolic pathway, we searched the libraries focusing on prostaglandin precursors. SWATH data raise the unexpected possibility that Cox-independent prostaglandins may be synthesized from lysophosphatidylcholine (LPC) intermediates. SWATH combined with classical genetics provides a powerful, untargeted approach to study lipids and generate novel hypotheses.

## 2. Results

A data-independent shotgun lipidomics workflow with SWATH acquisition was developed to perform comprehensive lipid analysis of wild-type and mutant *C. elegans*. This mass spectrometry technique generated an archive of all detectable precursor ions and their MS/MS fragment ions in samples within a 6 min run time. The MS and MS/MS spectra generated by the AB SCIEX 5600 triple-TOFMS instrument are high resolution (>15,000–30,000) and high mass accuracy (<5 ppm). Neutral lipids were extracted from staged one-day adult wild-type, *daf-1(m40)*, and *daf-2(e1370)* hermaphrodite worms shifted from 16 °C to 25 °C for 24 h. The *daf-2(e1370)* temperature-sensitive mutation causes reduced function at 25 °C [22]. Lipids were extracted from three 500 mg frozen worm pellets per genotype. Directly infused samples were acquired in positive and negative ion modes. During SWATH acquisition, precursor ion isolation windows of 1 Da width selected in Q1 are fragmented in the Q2 collision cell and the generated product ions are monitored at high resolution by TOF. The SWATH data include a 250 ms survey scan of TOFMS from *m*/*z* 200–1200, followed by MS/MS of 1000 ion windows. Four independent injections were conducted for each genotype, comprising 12 data libraries.

Internal standards were not included in this study due to several complicating factors (see Discussion). The goal of this method is to compare lipid species across genotypes. Samples were analyzed both in positive and negative ion mode with appropriate blanks between samples. There was no significant carry-over from samples. To assess the influence of sample preparation and the analytical device on variability, each sample was analyzed in biological and technical replicates. The reproducibility of the method was evaluated by monitoring responses of technical replicates of extracted worm samples. The intensities of ions between replicates were very similar (Supplemental Figure S1) with average coefficient of variation (CV) 5.67% in positive ion mode and 3.54% in negative ion mode. We conclude that the replicates are highly reproducible.

**Figure 2:**
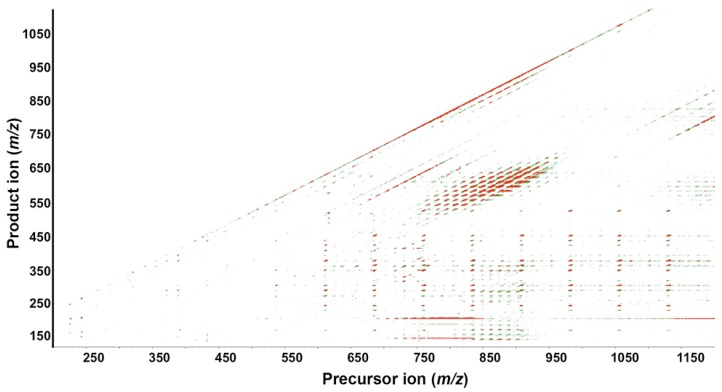
Contour plot of a wild-type extract acquired in positive ion mode. The fragmentation map shows precursor ions with mass/charge (*m*/*z*) 200–1200 on the *x*-axis and product ions with *m*/*z* 100–1100 on the *y*-axis. Each dot represents an individual lipid species, with color from green to red indicating relative increasing abundance (3rd dimension).

LipidView™ software 1.2 was used to search precursor- and fragment-ion masses against a lipid fragment database containing over 25,000 entries. To classify lipid species based on these database comparisons, the term annotated is used because absolute identification requires further analysis. Depending on structure, some lipids preferentially ionize in positive ion mode, whereas others preferentially ionize in negative ion mode. LipidView annotated 2817 lipid species covering a variety of classes, including 580 lipids in positive ion mode and 2237 in negative ion mode. The total independent species may be less because some lipids are detected in both ion modes. Figure 2 depicts a colored contour plot from a wild-type extract showing all precursor ions (*x*-axis) and product ions (*y*-axis) generated by SWATH acquisition in positive ion mode. It provides a snapshot of ion abundance. For example, the product ion *m*/*z* 184.0733 is a specific signature ion for phosphorylcholine moiety of glycerophospholipids and the precursor ion mass between *m*/*z* 700–850 shows strong ion intensity, as visualized by red color. Similarly, TAGs form a cluster of intense red peaks at precursor ion *m*/*z* 750–900 (*x*-axis) and product ion *m*/*z* 450–600 (*y*-axis). *C. elegans* synthesizes odd chain fatty acids [23,24]. In positive ion mode, a cluster of TAG ions between *m*/*z* 800–900 containing an odd chain fatty acyl chain (e.g., C17:0 or C19:0) was observed. Phosphatidylserine (PS), PI, and phosphatidic acid (PA) molecular species containing odd chain fatty acids were also observed in negative ion mode (see below).

Over 2500 ions (in positive and negative ion modes) were not annotated, perhaps reflecting software limitations, novel lipid species, or contaminating metabolites. The direct infusion method was unable to discriminate among isomers, including lipids with monomethyl branched chain fatty acids [23]. The lipids annotated by LipidView were imported into MarkerView™ 1.21 software, where trends could be visualized across replicates and groups. PeakView™ 1.2 software was used for targeted neutral loss and precursor ion scans, as well as viewing MS/MS spectra.

### 2.1. Characterization of Selected Lipids

**Figure 3:**
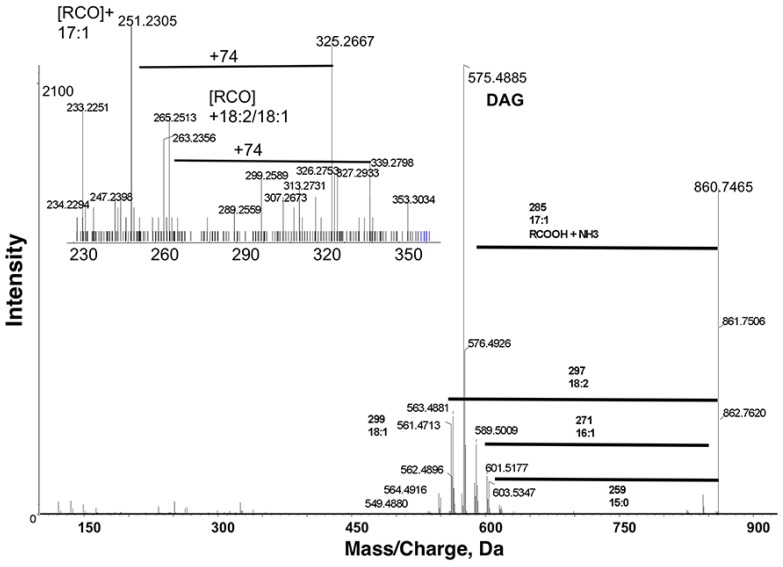
Product ion spectra obtained from MS/MS of *m*/*z* 860.7465 in positive ion mode. *m*/*z* 860.7465 [M+NH_4_]^+^ fragmented into a number of diacyl product ions, with the most intense product ion at *m*/*z* 575.4885 after neutral losses of ammonia (17 Da) and the fatty acid 17:1. The other less abundant product ions correspond to losses of 19:1, 19:0, 18:2 (shown), 18:1, 16:1 (shown), and 15:0 (shown). These spectra indicate that *m*/*z* 860.7465 corresponds to triacylglyceride (TAG) 51:3 + NH_4_.

We first sought to test whether SWATH could be used to discriminate among isobaric lipids. A prior targeted study using a data-dependent acquisition method characterized several TAG species in *C. elegans* extracts [9]. The most abundant TAGs contained multiple isobaric species with odd chain fatty acids. For example, TAG 51:3 was predominantly TAG 17:1/17:1/17:1, but also contained several other TAG species [9]. According to LipidView annotations, the most abundant TAGs in our extracts contain odd chain fatty acids. The prominent ion *m*/*z* 860.7465 in positive ion mode was annotated as TAG 51:3 + NH_4_. The ammonium adduct of TAG *m*/*z* 860.7465 [M+NH_4_]^+^ fragmented into a number of diacyl product ions, with the most intense product ion *m*/*z* 575.4885 after neutral losses of ammonia (17 Da) and the fatty acid 17:1 (Figure 3). The other product ions *m*/*z* 547.4559, 549.4880, 561.4713, 563.4881, 589.5009 and 601.5177 corresponded to losses of 19:1, 19:0, 18:2, 18:1, 16:1 and 15:0, respectively, likely due to less abundant isobaric TAG species. TAG molecular species can be proposed based on fatty acyl group neutral loss in MS/MS of *m*/*z* 860.7465. Since the intensity of diacyl product ions due to loss of 17:1 is the highest, this lipid was tentatively identified as TAG 17:1/17:1/17:1. Therefore, SWATH can successfully discriminate among isobaric TAGs in *C. elegans* extracts.

**Figure 4:**
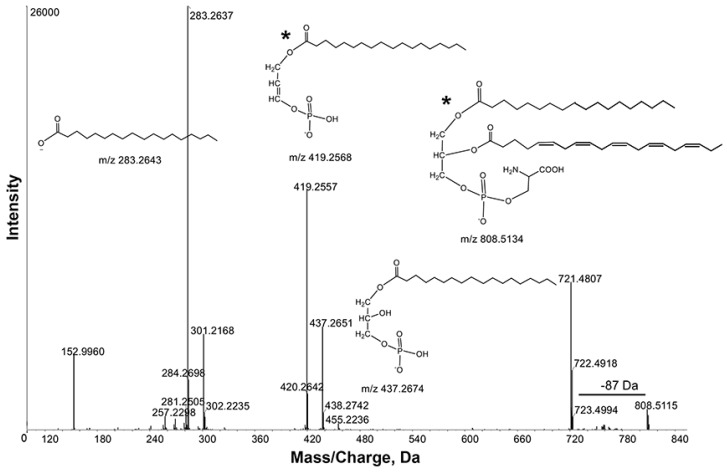
Product ion spectra obtained from MS/MS of *m*/*z* 808.5115 in negative ion mode. Key product ion structures with corresponding *m*/*z* are shown. These spectra identified *m*/*z* 808.5115 as Phosphatidylserine (PS) 38:5 (20:5_18:0). *, the *sn-1* and *sn-2* fatty acid positions indicated in the structures are arbitrary and cannot be inferred from the data.

Schwudke *et al.*, also characterized PS and PC species using respective neutral loss and precursor ion scans in positive ion mode [9]. We were able to characterize these lipids in both positive and negative ion modes. In positive ion mode, MS/MS of PS precursor ion [M+H]^+^ produces a product ion [M+H-185]^+^, resulting from loss of the phosphoserine moiety. In negative ion mode, the presence of the [M−H-87]^−^ product ion is characteristic of PS. The negative ion spectra are easier to interpret because they lack sodium adducts. Neutral loss scan Δ*m*/z 87.08 showed abundant PS species in the mass range *m*/*z* 700–900. The intense ion *m*/*z* 808.5115 contained product ions *m*/*z* 721.4807, 437.2651 and 419.2557 due to losses of [M-88], [M-88-301] and [M-88-283], respectively (Figure 4). These observations were further supported by product ions at *m*/*z* 301.2168 and 283.2637, corresponding to C20:5 and 18:0 fatty acyl chains. Thus, the ion *m*/*z* 808.5115 was tentatively identified as PS 38:5. Consistent with this interpretation, Schwudke *et al.*, identified PS 38:5 as the most abundant PS in wild-type extracts [9].

PCs generate an abundant product ion *m*/*z* 184.0733 in positive ion mode, corresponding to protonated phosphorylcholine. Precursor ion scan *m*/*z* 184.0733 detected a large cluster of PC species in the mass range *m*/*z* 700–850 (Supplemental Figure S2), consistent with the targeted approach [9]. In negative ion mode, PCs produce abundant [M-15]^−^ and [M+OAc]^−^ ions in the presence of ammonium acetate buffer [9,25]. We conclude that SWATH provides results comparable to targeted methods, yet with increased flexibility.

### 2.2. Lipid Changes in daf-1(m40) and daf-2(e1370) Worms

An advantage of SWATH is that it provides an objective starting point to compare lipids from different genotypes (or experimental conditions). A comparison of wild-type extracts to *daf-1* and *daf-2* mutant extracts showed widespread alterations in lipids, based on LipidView annotations. Principal component analysis (PCA), a statistical method to visualize variation and patterns in a dataset, of all ions observed in positive and negative ion modes indicated that the wild-type and two mutant strains cluster away from one another (Supplemental Figure S3A,B). Close clustering of the four replicates is also visible, indicative of high reproducibility. Below, we discuss selected lipid classes, including molecular species that changed significantly in *daf-1* and *daf-2* mutants. We include information on targeted precursor ion and neutral loss scans, as well as MS/MS spectra to help validate LipidView data. Comparisons between any two (or three) genotypes are possible, although we primarily focused on lipids altered in wild-type *versus* both *daf-1(m40)* and *daf-2(e1370)* extracts for simplicity. In Supplemental Table S1, we include data comparing wild-type extracts to either *daf-1(m40)* or *daf-2(e1370)* extracts. As a cautionary note, the lipid annotations in Supplemental Table S1 were supplied by LipidView software and were not verified. These data are a starting point for further investigation. The lipid libraries will be made publically available at the Common Fund Metabolomics Program Data Repository and www.mamillerlab.com. The raw data can be downloaded, imported into appropriate software, and analyzed according to specific needs of individuals.

#### 2.2.1. Triacylglycerides

**Figure 5:**
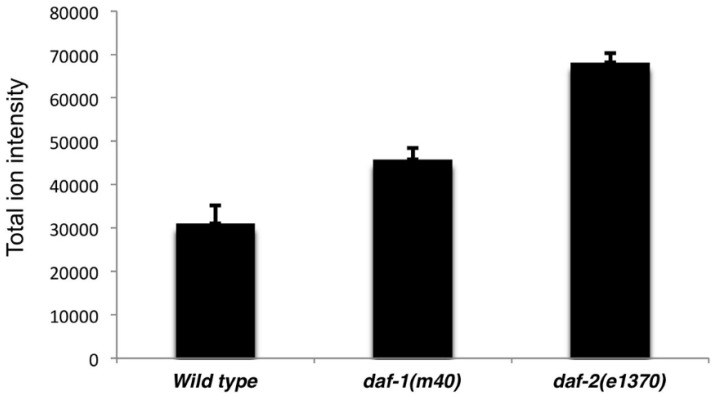
Total TAG lipidomes in wild-type and mutant extracts. TAG assignments are from LipidView.

Previous studies have shown that *daf-1* and *daf-2* mutants contain elevated TAGs, although the specific TAG species were not determined [17,18]. Using the lipid fragment database, LipidView annotated over 1000 potential TAG species. Compared to wild-type extracts, *daf-1(m40)* and *daf-2(e1370)* extracts showed ~1.5 and two-fold increases, respectively in total TAG molecular ion intensity (Figure 5, *p* < 0.005). Over 140 TAGs were significantly changed (*p* < 0.05) across the groups, with *daf-1* and *daf-2* mutants exhibiting distinct patterns. Table 1 shows the 20 TAGs with the lowest *p* values and fold changes. PCA indicates that the three genotypes are well separated. Moreover, close clustering is observed among replicates (Supplemental Figure S3C). TAGs annotations can be further investigated through MS/MS spectra reported in PeakView, as described for TAG 51:3 above (see Section 2.1). Therefore, SWATH detected the elevated TAG levels in *daf-1* and *daf-2* mutants with high reproducibility.

**Table 1:** Twenty TAG species with the most significant changes in wild-type extracts *versus* both *daf-1(m40)* and *daf-2(e1370)* extracts.

Lipid	*p* Value	Fold Change *
TAG 52:0 + NH_4_ (FA 20:5)	0.000007	0.56
TAG 55:5 + NH_4_ (FA 20:3)	0.00002	0.39
TAG 45:3 + NH_4_ (FA 15:1)	0.00009	0.01
TAG 46:2 + NH_4_ (FA 17:1)	0.00010	0.43
TAG 43:0 + NH_4_ (FA 15:0)	0.00017	0.09
TAG 53:5 + NH_4_ (FA 20:4)	0.00022	0.39
TAG 55:3 + NH_4_ (FA 19:1)	0.00022	0.51
TAG 46:7 + NH_4_ (FA 13:0)	0.00023	0.09
TAG 51:4 + NH_4_ (FA 17:2)	0.00027	0.56
TAG 56:3 + NH_4_ (FA 18:1	0.00029	0.48
TAG 53:4 + NH_4_ (FA 18:2)	0.00030	0.67
TAG 48:1 + NH_4_ (FA 15:1)	0.00034	0.35
TAG 54:3 + NH_4_ (FA 18:2)	0.00049	0.70
TAG 46:1 + NH_4_ (FA 17:0)	0.00050	0.46
TAG 57:4 + NH_4_ (FA 19:1)	0.00241	0.49
TAG 50:4 + NH_4_ (FA 17:2)	0.00275	0.42
TAG 52:4 + NH_4_ (FA 18:4)	0.00275	0.40
TAG 50:9 + NH_4_ (FA 19:1)	0.00308	0.35
TAG 51:3 + NH_4_ (FA 17:1)	0.00315	0.63
TAG 56:3 + NH_4_ (FA17:2)	0.00339	0.31

* wild type *versus* average of both *daf-1* and *daf-2* mutants.

#### 2.2.2. Diacylglycerides

Diacylglycerides (DAGs) are important signaling intermediates, often derived from PI or PC hydrolysis, as well as intermediates in TAG metabolism. LipidView identified DAGs in all three groups, although they were less abundant than TAGs. PCA shows that all groups are well separated (Supplemental Figure S3D). To validate selected LipidView assignments of molecular DAG species, PeakView was used to visualize MS/MS spectra. DAGs form an ammonium adduct [M+NH_4_]^+^ in positive ion mode when ammonium acetate is added into the mobile solvent. Upon MS/MS fragmentation, a precursor [M+NH_4_]^+^ DAG ion yields product ions with characteristic losses of 35 Da [loss of H_2_O + NH_3_] and the fatty acid together with ammonia [RCOOH + NH_3_], resulting in a monoacylglycerol-H_2_O fragment [26]. These features can be used for identification.

#### 2.2.3. Phosphatidylcholine and Phosphatidylserine

Glycerophospholipids include PC, PS, PA, PI, phosphatidylethanolamine (PE), and sphingomyelin (SM). These lipids are signaling intermediates and components of lipid bilayers. Analysis of glycerophospholipids depends on structural variation of head groups and fatty acyl chain lengths. PC and SM more readily ionize in positive ion mode, whereas PA, PE, PS, and PI more readily ionize in negative ion mode.

Based on LipidView assignments, both mutant extracts had increased levels of a large number of PC molecular species acetate adducts. 67 PCs were significantly changed (*p* < 0.05) across the groups. About 35% of them contained odd chain fatty acids. Table 2 shows the PC species with the most significant changes. PCA indicated the groups were well separated (Supplemental Figure S4A). We validated selected LipidView PC annotations using targeted approaches, briefly described in Section 2.1. Manual assignment of PC fatty acid moieties was obtained by their negative ion MS/MS spectra. 

**Table 2:** Twenty PC species with the most significant changes in wild-type extracts *versus* both *daf-1(m40)* and *daf-2(e1370)* extracts.

Lipid	*p* Value	Fold Change *
PC 38:1 + AcO (FA 19:1)	0.00003	1.36
PC 36:6 + AcO (FA 20:5)	0.00004	1.49
PC 36:2 + AcO (FA 19:1)	0.00005	1.58
PC 32:1 + AcO (FA 19:1)	0.00038	6.00
PC 34:2 + AcO (FA 17:1)	0.00045	1.61
PC 38:2 + AcO (FA 20:0)	0.00053	1.67
PC 34:4 + AcO (FA 18:3)	0.00088	1.31
PC 30:1 + AcO (FA 17:1)	0.00098	1.71
PC 34:5 + AcO (FA 18:3)	0.00107	1.37
PC 36:3 + AcO (FA 18:3)	0.00186	1.27
PC 36:3 + AcO (FA 18:0)	0.00226	1.30
PC 36:2 + AcO (FA 17:1)	0.00237	1.70
PC 36:0 + AcO (FA 15:0)	0.00253	1.76
PC 36:2 + AcO (FA 20:1)	0.00254	1.27
PC 32:0 + AcO (FA 20:0)	0.00272	1.41
PC 38:1 + AcO (FA 18:1)	0.00273	1.20
PC 36:2 + AcO (FA 18:0)	0.00307	1.31
PC 38:1 + AcO (FA 17:1)	0.00332	1.93
PC 38:4 + AcO (FA 20:1)	0.00404	1.46
PC 38:1 + AcO (FA 20:0)	0.0066	1.56

* wild type *versus* average of both *daf-1* and *daf-2* mutants.

LipidView identified over 70 PS species that were significantly changed (*p* < 0.05) across the groups. PS species containing odd chain fatty acids were commonly altered. Table 3 shows the 20 PS species with the most significant changes. PCA demonstrated good separation (Supplemental Figure S4B). PeakView was used to validate LipidView annotations, as described for PS 38:5 in Section 2.1.

#### 2.2.4. Phosphatidic Acid

LipidView identified over 50 PA species with significant changes in wild-type *versus* *daf-1* and *daf-2* mutant extracts (*p* < 0.01). *daf-2* mutants contained the highest levels of PAs. PCA showed a clear discrimination among the three groups (Supplemental Figure S4C). Targeted searches for PA species do not use a head group specific fragmentation ion, which is lacking [27]. As an anionic lipid, PA yields the deprotonated anion [M−H]^−^ and like all diacylglycerophosphates, it produces an MS/MS fragment ion *m*/*z* 153 [C_3_H_6_O_5_P]^−^ and ions corresponding to fatty acyl chains. For example, MS/MS fragmentation of the precursor ion *m*/*z* 719.4143 showed fragmentation patterns characteristic of PA molecules (Supplemental Figure S5). The losses due to [M-302] and [M-302-H_2_O] generated ions *m*/*z* 417.2396 and 435.2498, respectively. The presence of product ions *m*/*z* 152.9958, 301.2168, and 281.2483 enabled us to propose the tentative structure of *m*/*z* 719.4143 as PA 38:6.

**Table 3:** Twenty PS species with the most significant changes in wild-type extracts *versus* both *daf-1(m40)* and *daf-2(e1370)* extracts.

Lipid	*p* Value	Fold Change *
PS 34:4	0.00003	0.49
PS 34:3 (FA 17:0)	0.00004	0.62
PS 40:6 (FA 20:5)	0.00005	1.39
PS 30:0	0.00005	0.43
PS 32:2 (FA 17:0)	0.00012	0.67
PS 36:5 (FA 20:5)	0.00021	0.77
PS 34:2 (FA 18:2)	0.00024	0.70
PS 34:5	0.00030	0.41
PS 36:3 (FA 20:2)	0.00034	0.75
PS 38:2 (FA 17:1)	0.00036	1.54
PS 30:1	0.00038	0.73
PS 34:1 (FA 20:0)	0.00054	1.53
PS 38:3 (FA 20:2)	0.00064	0.60
PS 38:5 (FA 18:2)	0.00069	0.70
PS 34:0 (FA 17:0)	0.00085	0.78
PS 40:1 (FA 21:1)	0.00107	4.35
PS 38:5 (FA 20:2)	0.00173	0.51
PS 36:2 (FA 20:2)	0.00189	0.54
PS 40:1 (FA 19:0)	0.00233	1.66
PS 32:5 (FA 18:3)	0.00237	1.20

* wild type *versus* average of both *daf-1* and *daf-2* mutants.

#### 2.2.5. Phosphatidylinositol

**Figure 6:**
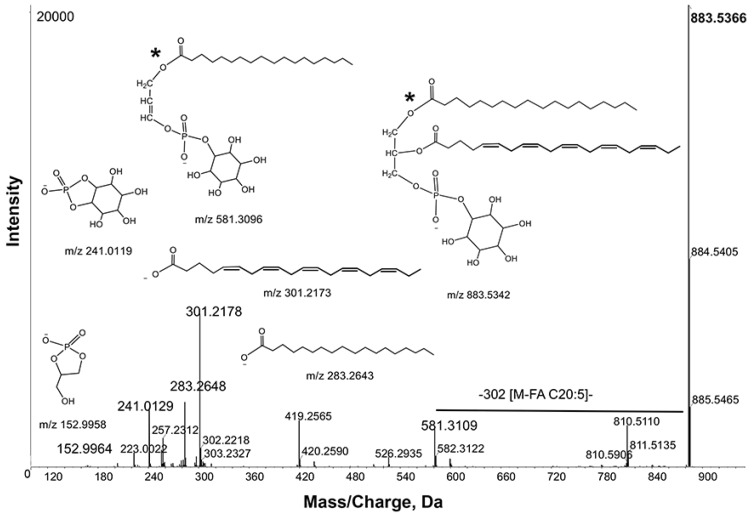
Product ion spectra obtained from MS/MS of *m*/*z* 883.5366 in negative ion mode. Key product ion structures with corresponding *m*/*z* are shown. These spectra identified *m*/*z* 883.5366 as PI 20:5_18:0).

PI species were significantly changed among the groups. The 10 species with most significant changes are listed in Table 4. In general, PIs were more abundant in the two mutants than in the wild type. PCA showed good separation (Supplemental Figure S4D). Targeted PI searches can be done in positive and negative ion modes. When PIs are analyzed in positive ion mode in the presence of ammonium acetate, they appear as ammonium adducts [M+NH_4_]^+^. In negative ion mode, deprotonated molecular ion [M−H]^−^ *m*/*z* 883.5366 yielded fragment ions *m*/*z* 581.3109 and 599.2717 corresponding to neutral losses of 302 (20:5) and 284 (18:0), respectively. These losses resulted in product ions *m*/*z* 301.2178 and 283.2648. The product ion *m*/*z* 419.2565 can be generated with loss of 162 (inositol-H_2_O) from the ion *m*/*z* 581.3109 (Figure 6). The product ions corresponding to the inositol head group and phosphodiester derivative of glycerol are *m*/*z* 241.0129 and 152.996, respectively. Based on these observations, the ion *m*/*z* 883.5366 was tentatively identified as PI 38:5.

**Table 4:** Ten PI species with the most significant changes in wild-type extracts *versus* both *daf-1(m40)* and *daf-2(e1370)* extracts.

Lipid	*p* Value	Fold Change *
PI 36:4 (FA 18:2)	0.00011	1.29
PI 36:5 (FA 18:2)	0.00028	1.32
PI 32:0 (FA 17:0)	0.00037	0.69
PI 36:2 (FA 19:1)	0.0005	1.36
PI 36:5 (FA 18:3)	0.00053	1.60
PI 36:4 (FA 18:3)	0.00066	1.35
PI 34:2 (FA 20:2)	0.00069	0.50
PI 36:6 (FA 18:3)	0.00073	1.34
PI 36:0 (FA 18:0)	0.0009	1.29
PI 28:0 (FA 16:0)	0.00152	0.70

* wild type *versus* average of both *daf-1* and *daf-2* mutants.

*, the *sn-1* and *sn-2* fatty acid positions indicated in the structures are arbitrary and cannot be inferred from the data.

#### 2.2.6. Other Lipids

In addition to the lipids discussed above, LipidView identified changes in lipids belonging to other classes. For instance, SM and *N*-Acylphosphatidylethanolamine (NAPE) species were consistently altered among the groups. NAPE species, in particular, exhibit low abundance and we could not confirm the identity of these lipids with MS/MS. Altering extraction conditions or mass spectrometry parameters could improve detection. We also investigated novel lipids, which can be discovered using neutral loss or precursor ion scans. For example, precursor ion scan *m*/*z* 184.0733 in positive ion mode for choline-containing lipids yielded a cluster in the mass range *m*/*z* 1100–1200. Searching the database with precursor ions *m*/*z* 1184.7933 and 1198.7940 yielded no results, and interpretation of their product ions indicated these lipids are novel PCs. MS/MS of *m*/*z* 1198.7940 showed product ions *m*/*z* 184.0749 and *m*/*z* 124.9986, which are characteristic of PCs (Supplemental Figure S6). The presence of ion *m*/*z* 784.5849 in the product ion spectrum of *m*/*z* 1198.7940 indicates that this ion did not result from aggregation.

### 2.3. Delineating Novel Lipid Metabolism Pathways

Above we described untargeted and targeted analyses of SWATH libraries, focusing on selected lipid classes. The comparative method also provides an objective opportunity to investigate lipid metabolism pathways. SWATH might be used to identify candidate lipid intermediates based on empirical data from other sources. The Cox-independent prostaglandin pathway was used as an example. *daf-1* and *daf-2* are both required for Cox-independent prostaglandin synthesis, but act at different steps (Figure 1B). *daf-2(e1370)* disrupts prostaglandin precursor transport to oocytes [15], whereas *daf-1(m40)* inhibits a downstream biochemical step, likely through reduced expression of an enzyme [16]. When enzyme activity is blocked, a strong increase in substrate abundance results. For example, knockout of the omega-3 desaturase enzyme *fat-1* causes a 12.8 fold increase in the substrate AA or 20:4 [28]. Relative to the wild type, *daf-1* mutants are predicted to contain strongly increased levels of an unknown prostaglandin intermediate, which is reduced in *daf-2* mutants.

**Figure 7:**
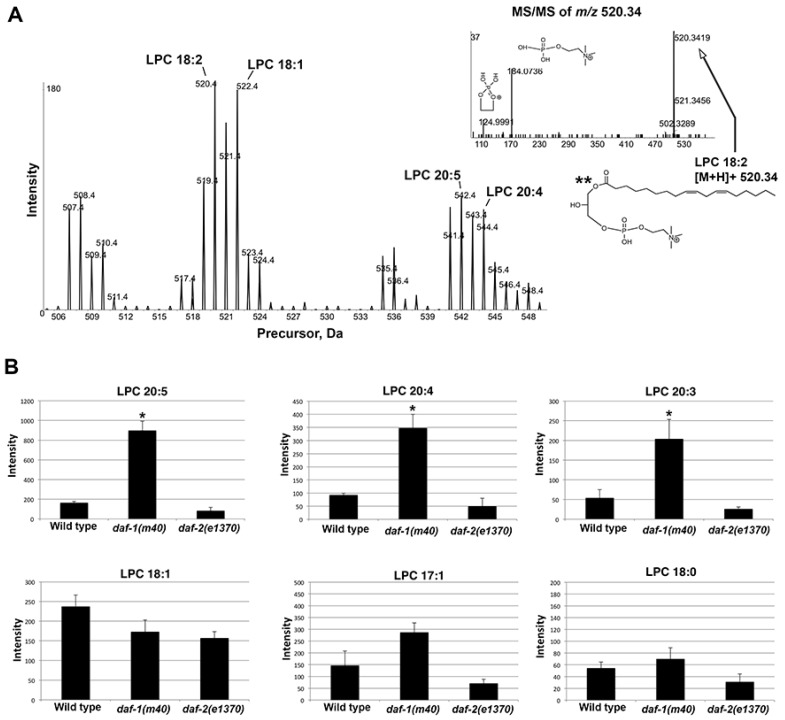
LPC species in wild-type and mutant extracts. (**A**) Precursor ion scan *m*/*z* 184.0733 in positive ion mode detects a series of LPCs between *m*/*z* 505–550. MS/MS in the inset (upper right) shows LPC 18:2 product ion spectra with corresponding structures. (**B**) Selected LPCs quantified from precursor ion scan *m*/*z* 184.0733 in positive ion mode. *C. elegans* prostaglandins are synthesized from dihomo-gamma-linolenic acid (20:3), AA (20:4), and EPA (20:5).

First, using the same staged nematode growth conditions as those used for SWATH, we confirmed that *daf-1(m40)* hermaphrodites have reduced prostaglandin synthesis. Consistent with a prior study [16], LC-MS/MS operated in multiple-reaction monitoring (MRM) mode showed strong reductions in F-series prostaglandins derived from DGLA (20:3), AA (20:4), and EPA (20:5) (Supplemental Figure S7). Next, we used MarkerView to search the SWATH libraries for all lipids specifically altered in *daf-1* mutants *versus* the other two strains. MarkerView identified LPC 20:5 and LPC 20:4 as two lipids with the most robust changes. PCA showed clear LPC separation among the genotypes (Supplemental Figure S8). To validate these data, we used precursor ion scan *m*/*z* 184.0733 in positive ion mode. A series of ions was detected between *m*/*z* 505–550. MS/MS confirmed LPC identity (Figure 7A). LPC 20:3, LPC 20:4, and LPC 20:5 increased by 3–6 fold in *daf-1* mutant *versus* wild-type extracts (Figure 7B). LPCs containing monounsaturated and saturated fatty acyl chains were much less affected, although saturated LPCs were close to the detection level (Figure 7B). LPCs in *daf-2* mutants were reduced by approximately 50% compared to the wild type (Figure 7B). We conclude that untargeted and targeted SWATH approaches identified LPCs containing prostaglandin precursors as specifically increased in *daf-1* mutants.

Prostaglandin precursors are transported in yolk lipoprotein complexes, which primarily contain C20 PUFAs in PC, PE, and free forms [21]. Although MarkerView did not detect statistically significant changes in LPE and free PUFAs, we considered the possibility that low levels might confound LipidView assignments. First, we used PeakView to evaluate LPEs manually. Neutral loss scan *m*/*z* 141.00 conducted in positive ion mode identified LPE species between *m*/*z* 460–510 in *daf-1* mutants. Among the three groups, LPEs containing prostaglandin precursors showed the same trend as LPCs, except LPE levels were close to the detection limit. For quantification of free PUFAs, we developed an LC-MS/MS method operated in MRM mode. MRM analysis showed that free 20:4 and 20:5 levels were increased by 20.2% and 60.3%, respectively in *daf-1(m40)* extracts compared to wild-type extracts (Supplemental Figure S9). These data indicate that LipidView may miss less abundant or less ionizable lipids detectable using targeted approaches. In summary, comparative SWATH lipidomics identified LPCs and possibly LPEs containing PUFAs as strongly up-regulated in *daf-1* mutants. Other lipids containing prostaglandin precursors were much less affected (*i.e.,* free 20:4 and 20:5) or unaffected.

*, *p* < 0.005. **, the *sn-1* fatty acid position indicated in the structure is arbitrary (*sn-1 vs.* *sn-2*) and cannot be inferred from the data.

## 3. Discussion

A key feature of this study is the combined use of data-independent SWATH and genetic manipulation to investigate lipid biology. In *C. elegans*, mutational or RNA-mediated interference screens can be designed to identify enzymes and other lipid regulators, based on phenotypes resulting from pathway dysfunction. For example, TGF-β pathway components were identified in screens for sperm motility defects, which occur when Cox-independent prostaglandin synthesis is disrupted [15,16]. CRISPR/Cas9 and other genome-editing technologies are available to modify or delete gene products of interest [29,30,31]. The comparative SWATH approaches outlined here are well suited for discovery of lipid biomarkers, enzyme substrates, and metabolic pathways.

We focused on two well-studied mutant strains to evaluate method effectiveness. Disrupting insulin and TGF-β signaling causes overlapping phenotypes, but the molecular mechanisms are distinct [13,14]. Similarly, the mutants have different lipid profiles. These profiles could be used to identify downstream lipid mediators or develop “lipid signatures” for assessing signaling activity. Previous studies have demonstrated elevated TAG levels in *daf-1* and *daf-2* mutants. Our SWATH method identified increases in total TAG ion intensity in the mutants, as well as identified the individual molecular TAG species. SWATH also provided an unexpected model for Cox-independent prostaglandin metabolism: LPCs and other lyso glycerophospholipids may be key intermediates. As shown in the Figure 1B example, we can predict that biochemical step A occurs via phospholipase activity, producing LPC 20:4. During step B, TGF-β signaling promotes expression of an enzyme that acts on LPC 20:4. The number of steps between LPC 20:4 and PGF2 isomers are unknown and could be large. This testable model is consistent with failure of dietary 20:4 supplementation to rescue *daf-1(m40)* sperm motility defects [32]. Future genetic screening and SWATH could help identify enzymes and substrates, providing a framework for the pathway. Stable isotope labeling studies could also be used to trace pathway flux.

In targeted approaches, internal standards are often included to account for variation in lipid extraction efficiency, ionization efficiency, *etc.* However, the untargeted nature of SWATH makes the choice of internal standards difficult. Lipids containing odd-chain fatty acids or stable isotopes are used as internal standards in mammalian studies. *C. elegans* synthesizes odd-chain fatty acids, so these inexpensive options are not good choices. More problematic, lipids containing stable isotopes are expensive and unavailable for most lipid classes. With the present limited selection, we suggest excluding internal standards for *C. elegans* SWATH. To avoid confounding issues, multiple independent replicates should be conducted to assess variability. Lipids exhibiting high variation among replicates or replicates with high coefficient of variation should be interpreted with extreme caution. When a set of altered lipids is chosen for further studies, a targeted approach using the best available internal standards can be developed. It is important to remember that SWATH provides an objective method for discovery. SWATH is not a substitute for MRM or other methods capable of absolute quantification.

There are shortcomings to the SWATH approach. First, the coverage of lipids is not complete. Although SWATH detected several thousand species, extraction conditions and mass spectrometer settings, such as collision energy will influence coverage. For example, our neutral lipid extraction method is not efficient at extracting oxidized lipids and multiple conditions should be explored. Second, LipidView does not always accurately annotate lipid species, which should be confirmed through MS/MS interpretation. Third, as mentioned above, SWATH does not provide absolute quantification. Despite these shortcomings, the advantages are powerful. In particular, the ability to retrospectively search SWATH libraries is important for lipid identification and biological discovery. Changes in a specific lipid species between two samples might suggest changes in precursors or related lipids. As new hypotheses form, they can be tested across all generated libraries. SWATH libraries can be searched by any researcher with appropriate software, obviating the need to repeat sample preparation and analysis. In genetic models where tissue is limiting, a well-constructed library provides a valuable resource for the entire community.

## 4. Experimental Section

### 4.1. Chemicals

The standards AA and eicosapentaenoic acid (EPA) were purchased from Cayman Chemical Co. (Ann Arbor, MI, USA). All HPLC solvents and reagents were purchased from Fisher Scientific Co. (Norcross, GA, USA) and were of HPLC grade.

### 4.2. Sample Preparation and Extraction

N2 Bristol (wild type), DR40 *daf-1(m40)* *IV*, and CB1370 *daf-2(e1370) III* *C. elegans* were grown at 16 °C on nematode growth media and fed with NA22 *E. coli* bacteria. Worms were synchronized to the L1 stage using an egg preparation with minor modifications [20]. About 60,000 L1 larvae were grown on each of forty 15 cm seeded plates for 2–3 days until adulthood. Cultures were supplemented with concentrated bacteria as needed to prevent starvation. Gravid worms were collected and washed with M9 buffer. 500 mg aliquots of cleaned synchronized wild-type and mutant worms were stored in 5-mL polypropylene tubes at −80 °C for lipid extraction.

To reduce oxidation during and after extraction, 0.005% butylated hydroxytoluene was added to organic solvents. Three 0.5 g aliquots of each genotype were extracted separately. 1 mL of 0.5 mm diameter Ceria stabilized zirconium oxide beads, 1 mL of methanol, and 500 μL of chloroform were added to each tube. Worms were homogenized in a Bullet Blender 5 at speed 10 for three minutes [20]. 500 μL of chloroform, 400 μL of water, and 75 μL of 2M formic acid were added to reach pH3.5. After vortexing for thirty seconds, the tubes were centrifuged at 1000× *g* for one minute. The lower chloroform phase was transferred to a clean Sigmacote-coated glass tube. 2.5 mL of chloroform was added to the remaining aqueous solution. After vortexing for thirty seconds, the tube was centrifuged at 1000× *g* for one minute and the lower chloroform phase was again transferred. The pooled chloroform phases from each sample were stored at −20 °C overnight. The next day, the remaining milky aqueous top layer was discarded. The chloroform phase was transferred to a labeled Teflon-lined ½-Dram glass vial using a new Pasteur pipet. In a chemical hood, the chloroform fraction was evaporated to dryness under a gentle flow of N_2_ gas.

### 4.3. Lipid Analysis

Worm extracts were analyzed by direct infusion analysis. Approximately 100 μL of diluted lipid extract in methanol:chloroform (2:1 v/v) with 5 mM Ammonium acetate was delivered to the source by isocratic flow at 7 μL/min using a 500 μL Hamilton Gas Tight Syringe. For syringe cleaning, prior to and in between samples, the direct infusion syringe was cleaned with multiple solvents. The solvent wash steps included two flushes with 100% methanol, two flushes with 100% acetonitrile, two flushes with 100% isopropyl alcohol, and two flushes with 100% direct infusion solvent. Calibration standards for direct infusion analyses were provided by SCIEX. The APCI Positive Calibration Solution (Part # 4460131) and APCI Negative Calibration Solution (Part# 4460134) were utilized.

### 4.4. Mass Spectrometry Analyses

Positive and negative ion MS and MS/MS were carried out on a Triple TOFTM 5600 System (SCIEX, Concord, ON, Canada). An MS experiment was carried out from *m*/*z* 200–1200. Initially, one 250 msec high resolution TOF scan was acquired. Next, a series of 100 msec high sensitivity product ion scans were acquired per one Dalton (1 *m*/*z*) mass starting at *m*/*z* 200 and increasing in one size steps through *m*/*z* 1200. The collision energy was set to 35 V, curtain gas to 20.00, GS1 and GS2 to 15.00, spray voltage to 5500.00 (positive ion mode)/4500 (negative ion mode), and temperature to 400 °C. The total time to carry out the entire experiment was 6 min.

### 4.5. Data Analysis

The acquired TOF MS and MS/MS data were processed with LipidView™ 1.2 software (SCIEX, Concord, ON, Canada). LipidView assigns lipid identities based on a fragmentation database. To further investigate ions and confirm selected identities, neutral loss or precursor ion scans were carried out using PeakView™ 1.2 software (SCIEX, Concord, ON, Canada). The mass tolerance window for processing was set at 5 mDa and the peaks in MS/MS scans greater than signal-to-noise of 3 were considered. Identification of individual lipid species from LipidView assignments was based on mass accuracy (<5 ppm) and MS/MS spectra obtained from PeakView. High resolution TOFMS provided the accurate mas of precursor ions. MarkerView 1.21 software (SCIEX, Concord, ON, Canada) was used to visualize trends across replicates and groups. Principal component analysis was performed to visualize the similarities and differences within and between groups, based on LipidView assignments.

### 4.6. LC-MS/MS Analysis of Free AA, EPA, and F-Series Prostaglandins

Quantification of free AA and EPA was performed using a system consisting of an Eksigent Micro-LC 200 and an API 4000 (SCIEX, Concord, ON, Canada) triple quadrupole mass spectrometer. Chromatography was performed using a Luna 3u C18(2) 100A 0.5 × 50 mm column (Phenomenex, CA, USA). The flow rate was 40 μL/min, with the gradient starting with 45% B (acetonitrile containing 5 mM ammonium acetate) to 95% B over 15 min and maintaining B for 1 min. At 17 min there was a linear decrease 95% to 45% B. Total run time was 20 min. Mass transitions for AA were 303/59 and for EPA were 301/121, 301/135, and 301/241. Calibration standards for AA and EPA (50, 25, 10, 1, 2, 0.1 and 0.05 ng/mL) and *C. elegans* lipid extracts were prepared in methanol:water (8:2 v/v). AA–d8 was used as an internal standard. F-series prostaglandins were extracted and analyzed as previously described [16,20]. Worms were synchronized and cultured as described in Section 4.2.

## 5. Conclusions

MS/MS^ALL^ SWATH technology created comprehensive lipid data libraries from *C. elegans* extracts that can mined retrospectively using untargeted and targeted approaches. A comparative strategy identified numerous changes in lipid species among wild type and mutant worms. Examples were included for validating untargeted data and identifying isobaric species. Finally, and as proof of principle, we used SWATH to investigate an incompletely characterized lipid metabolism pathway. In summary, comparative SWATH lipidomics is a powerful, objective method to identify candidate biomarkers, pathway intermediates, and other lipids important for biological function.

## Supplementary Files

Supplementary File 1
