# Supplementary material for: Comparative Lipidomics of Caenorhabditis elegans Metabolic Disease Models by SWATH Non-Targeted Tandem Mass Spectrometry
Source: Metabolites. 2015 Nov 11;5(4):677–96. doi: 10.3390/metabo5040677 (PMC4693190; doi:10.3390/metabo5040677)
Supplement: Supplementary File 1 [file metabolites-05-00677-s001.zip › Figures S1-S9.docx]

**OPEN ACCESS**

***metabolites***

**ISSN 2218-1989**

www.mdpi.com/journal/metabolites/

Supplementary Materials


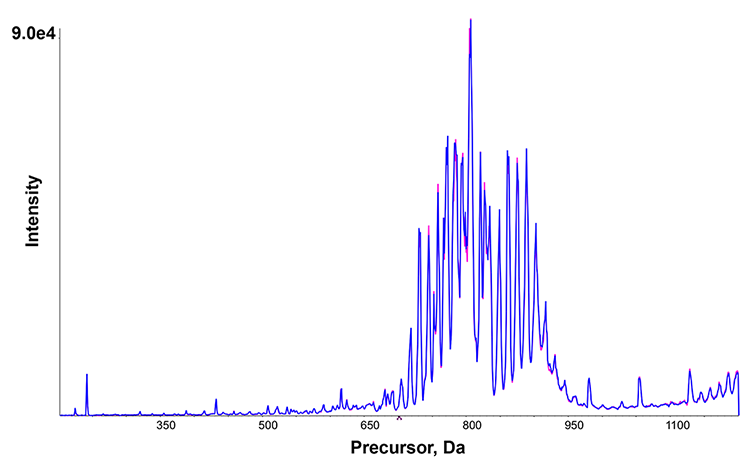


**Figure S1.** Total ion plots in positive ion mode from two replicates of wild-type worm extracts. The replicates in blue and pink are overlaid. Among all replicates, the average coefficient of variation was 5.67% in positive ion mode and 3.54% in negative ion mode.


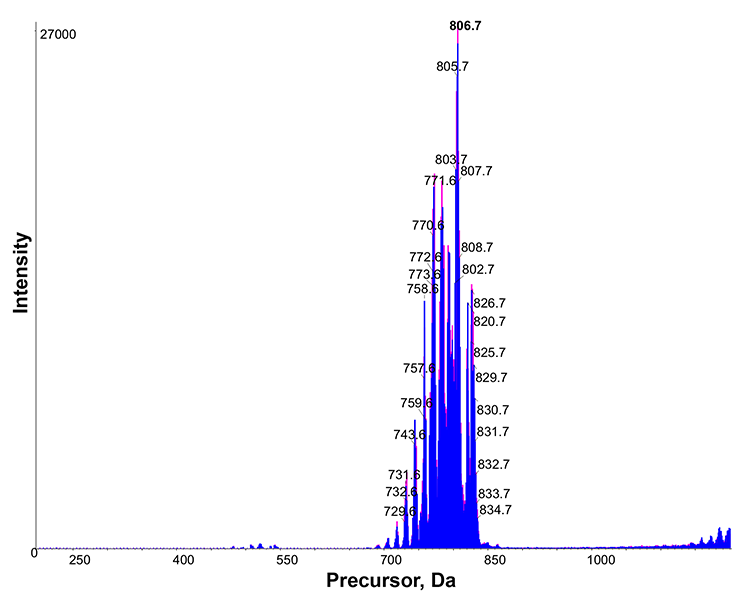


**Figure S2.** Precursor ion scan *m/z* 184.0733 in positive ion mode of wild-type extracts. Two replicates in blue and pink are overlaid. A large cluster of PC species in the mass range *m/z* 700–850 is observed. A similar cluster was observed in Schwudke *et al.* [9], who used a
data-dependent targeted strategy to detect PCs in *C. elegans*. Also notice a small cluster of LPCs in the mass range *m/z* 450–550 (see Figure 7) and unknown lipids containing phosphorylcholine in the mass range *m/z* 1100–1200 (see Figure S6).


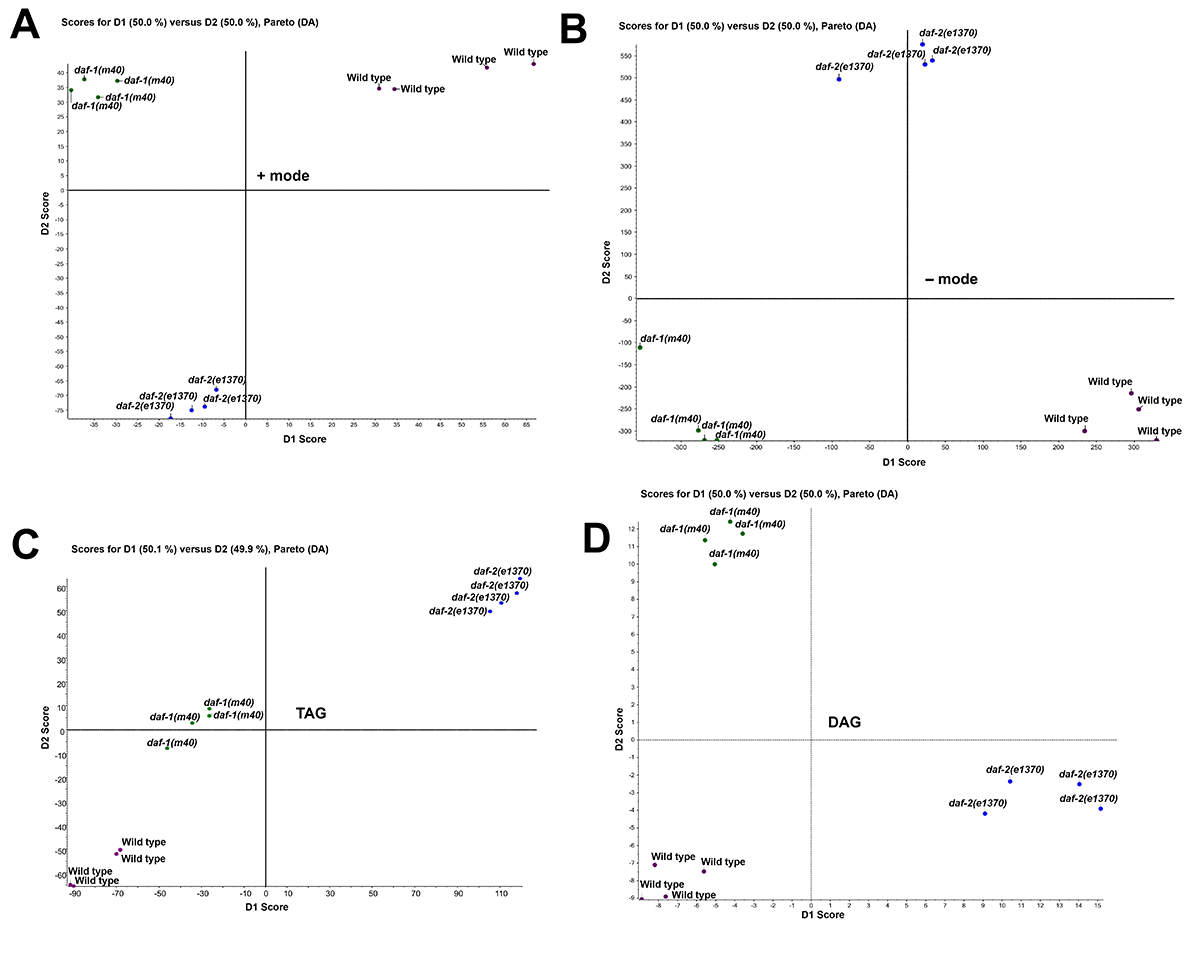


**Figure S3.** PCA score plots from wild-type and mutant extracts. These plots provide a visual distribution of lipid features. Close clustering of replicates is a strong indicator of reproducibility. Shown are lipids in positive ion mode (**A**); lipids in negative ion mode (**B**); TAGs (**C**); and DAGs (**D**).


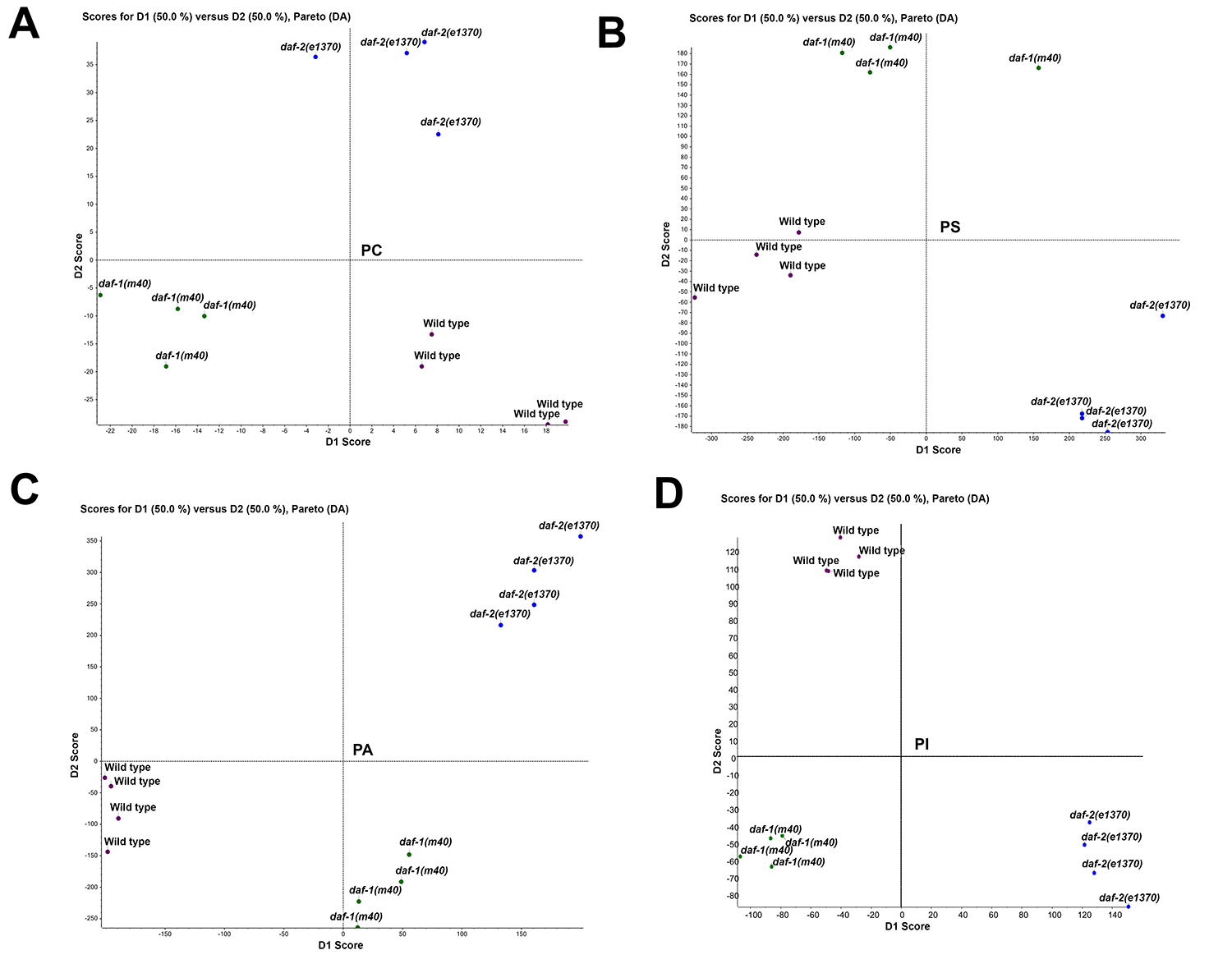


**Figure S4.** PCA score plots of PC (**A**); PS (**B**); PA (**C**); and PI (**D**) from wild-type and mutant extracts.


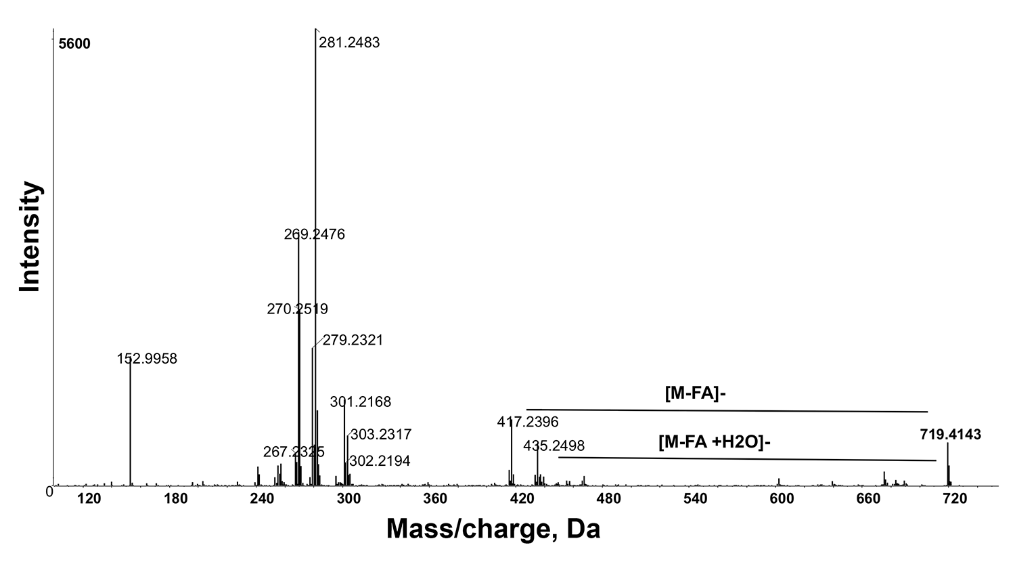


**Figure S5.** Product ion spectrum obtained from *m/z* 719.4143 in negative ion mode. Losses of [M-302] and [M-302-H2O] generated ions *m/z* 417.2396 and 435.2498, respectively. Furthermore, product ions *m/z* 152.9958, 301.2168, and 281.2483 support the proposed structure PA 18:1_20:5.


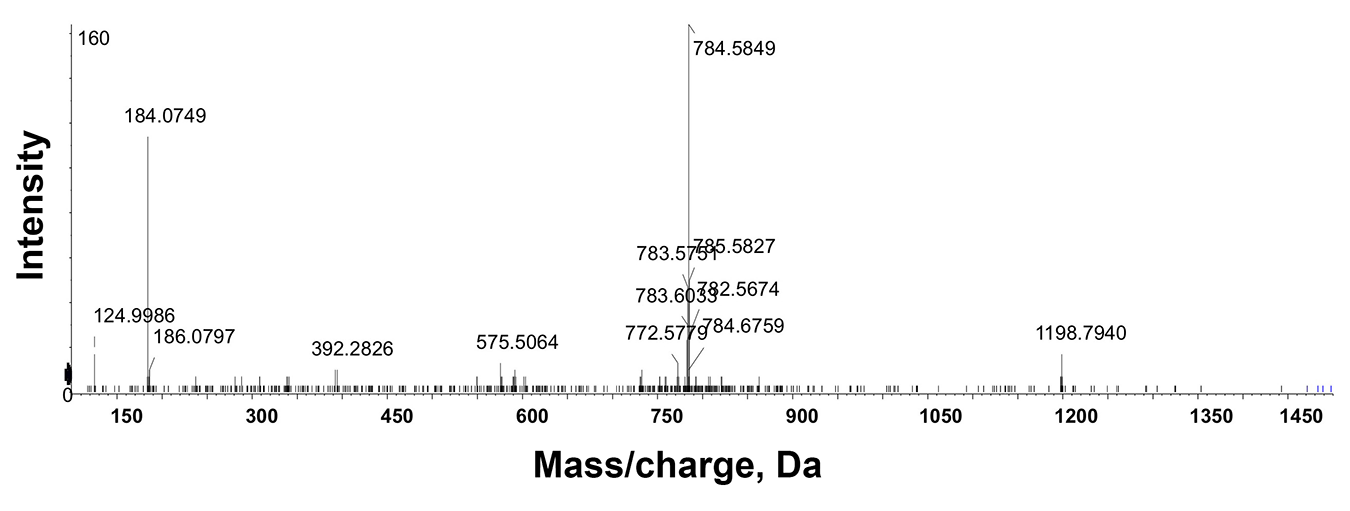


**Figure S6.** Product ion spectrum obtained from *m/z* 1198.7940 in positive ion mode. The product ions *m/z* 184.0749 and *m/z* 124.9986 suggest that this unknown lipid contains phosphorylcholine.


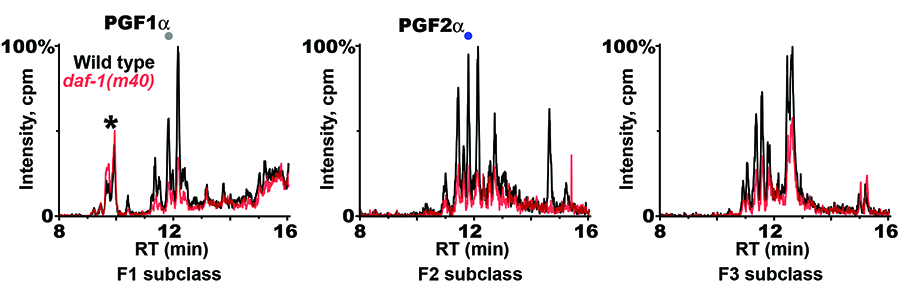


**Figure S7.** F-series Prostaglandins in wild-type and *daf-1(m40)* mutant extracts. MRM chromatograms are shown. Staged hermaphrodite worms were grown at 16 °C and shifted to 25 °C for 24 h. The F1 class was detected with MRM mass transition *m/z* 355/311, the F2 class with *m/z* 353/193, and the F3 class with *m/z* 351/193 [16,20]. Each peak indicates a prostaglandin isomer [20]. CePGF1 and CePGF2, which co-elute with respective PGF1α and PGF2α standards (indicated dots), were quantified in McKnight *et al.* [16]. *, lipid that is not a prostaglandin (note that it is not reduced in *daf-1(m40)* mutants). Approximately 75%–80% of prostaglandins derives from the female germ line, whereas the remainder derives from other tissues [20]. PGF2α d9 was used as an internal standard.


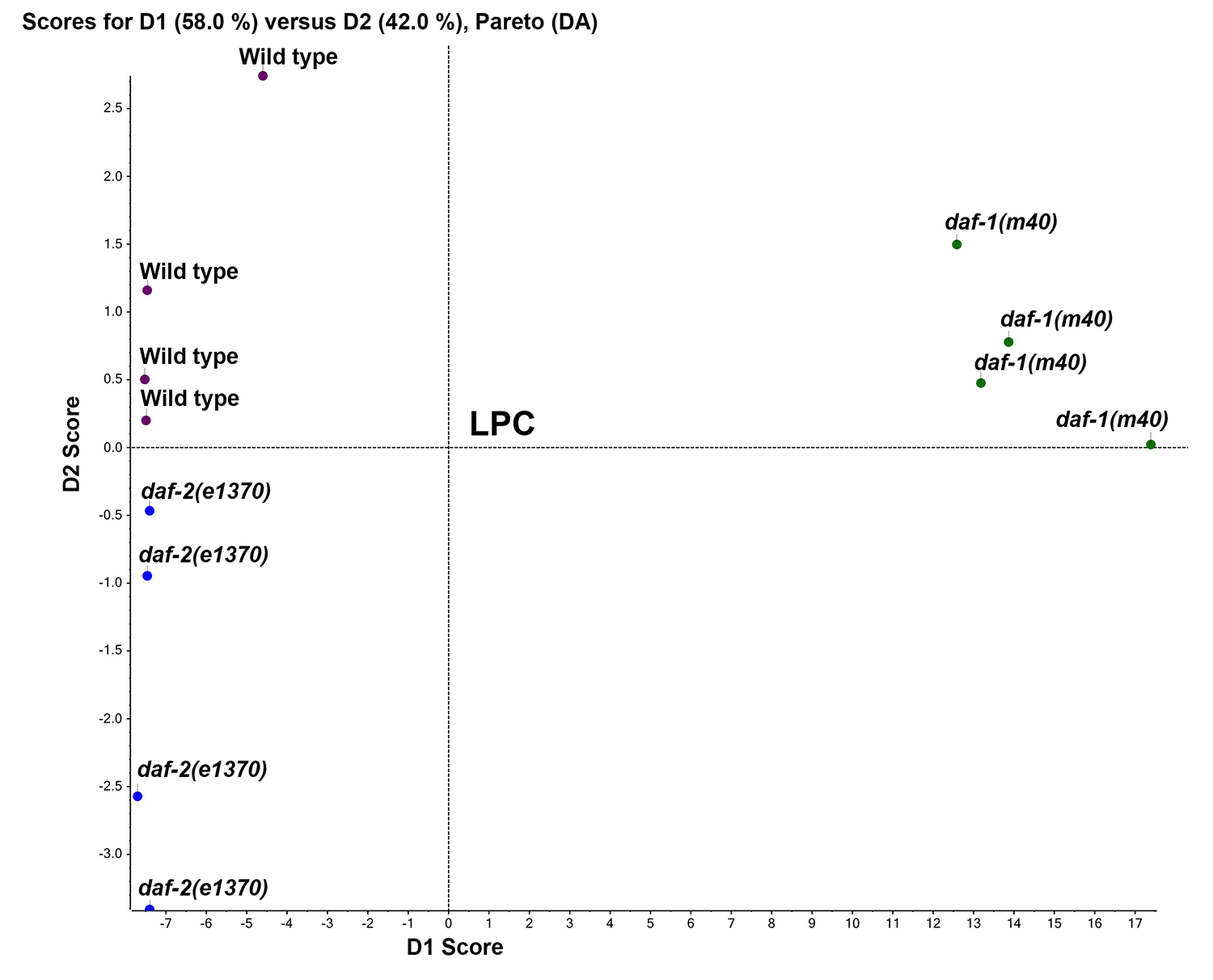


**Figure S8.** PCA score plot of LPCs from wild-type and mutant extracts.


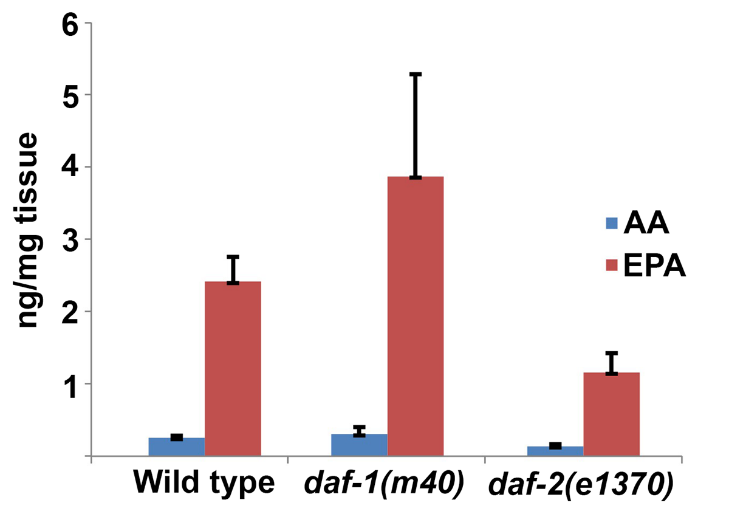


**Figure S9.** MRM based quantitative analysis of free arachidonic acid (AA) and eicosapentaenoic acid (EPA) in wild-type and mutant extracts. AA–d8 was used as an internal standard.

© 2015 by the authors; licensee MDPI, Basel, Switzerland. This article is an open access article distributed under the terms and conditions of the Creative Commons Attribution license (http://creativecommons.org/licenses/by/4.0/).
